# A systematic study of pulse and pulse reverse plating on acid copper bath for decorative and functional applications

**DOI:** 10.1038/s41598-022-22650-x

**Published:** 2022-10-28

**Authors:** Elena Mariani, Walter Giurlani, Marco Bonechi, Vincenzo Dell’Aquila, Massimo Innocenti

**Affiliations:** 1 Dipartimento di Chimica, Università degli Studi di Firenze, Via della Lastruccia 3, 50019 Sesto Fiorentino, Italy; 2 INSTM, Consorzio Interuniversitario Nazionale per la Scienza e Tecnologia dei Materiali, Via G. Giusti 9, 50121 Florence, Italy; 3 Eco-Tech Finish s.r.l., Z.I. San Zeno, Strada C 27, 52100 Arezzo, Italy; 4 CNR-ICCOM, Istituto di Chimica dei Composti OrganoMetallici, Via Madonna del Piano 10, 50019 Sesto Fiorentino, FI Italy; 5 CSGI, Center for Colloid and Surface Science, Via della Lastruccia 3, 50019 Sesto Fiorentino, Italy

**Keywords:** Chemical engineering, Electrochemistry, Materials chemistry

## Abstract

Today industrial electroplating is mainly carried out using direct current even if the use of modulated currents could offer greats opportunities. Adjusting the amplitude and length of the current’s pulses it is possible to control grain size, porosity and homogeneity of the deposits; the use of modulated currents could also decrease the environmental impact of deposition processes as they require a much lower percentage of organic additives. The aim of this work is to assess, through both theoretical and experimental investigation, how the deposition parameters affect the various characteristics of the deposit. We used a commercial acid copper bath for the depositions performing both pulse and reverse pulse sequences. The coatings have been characterised by estimating the deposition yield, homogeneity, hardness and reflectivity. Using pulsed currents, we obtained shinier and brighter films respect to those produced with stationary currents; the deposition efficiency was also improved. Bipolar currents, on the other hand, favour more homogeneous deposits over the entire deposition area, and are less affected by the edge effect.

## Introduction

Electroplating of copper is one of the most commonly used plating processes for both functional and decorative applications^1,2^. Pulsed (PC) and pulsed-reverse current (PRC) plating is widely used in electronics for printed circuit boards^3^, but also for the deposition of alloys^4^, composite materials^5^ and semiconductors^6,7^. On the contrary, only direct current (DC) is used in the decorative field^2^. Electroplating with modulated currents has been mostly dormant and only occasionally used for copper cyanide deposits, due to the levelling effects recognised in the early 1940s and 1950s^8^. Numerous studies on pulsed current deposition date back to the 1960s and 1970s^1^. At that time the rectifiers were not yet able to generate very high and their prohibitive cost hampered the interest in this otherwise promising technique. Today the technology is mature and there are rectifiers on the market capable of supplying high currents with sufficiently short pulses with respect to the electrodeposition phenomena occurring at the electrode.

Although it was recognised that in deposits of gold, or its alloys, the best morphology was obtained under pulsed or reverse pulsed current conditions^8^, the breaking point occurred as the importance, and value, of gold as a commodity, used in many electronic applications, grew. There is also a significant increase in the ductility of these deposits and an increase in electrical conductivity and density, as well as a decrease in porosity. Decrease in porosity in practice means that a thinner gold deposit can be applied and still pass porosity tests, resulting in a significant cost saving. Today the use of modulated currents is restricted to electronic applications in the production of printed circuits for deposition in narrow channels^8^. In the literature there are few studies concerning this topic for decorative applications^9^. In this industrial sector modulated currents could help in the reduction of organic brightening agents and of deposition unevenness with the consequent environmental and economic benefits^10^.

Pulse plating emerged as a new technique for the deposition of metals and alloys, with numerous theoretical contributions and industrial applications^11^. The advent of modern electronics and microprocessor control has allowed great flexibility in programming the waveform of the applied current, precisely because it is possible to control the composition and thickness, in atomic order, of the deposited films simply by adjusting the amplitude and length of the pulses^8,12–15^.

Waveforms can be divided into two main groups: unipolar pulses, where all pulses have one sign, and bipolar pulses, where anodic and cathodic pulses are combined. The simplest case of unipolar and bipolar pulses are the pulse and the reverse pulse, respectively. To characterise a direct current, it is sufficient to know the current density; for a pulsed current, the knowledge of three parameters is required: the cathode peak current density of the pulse, j_c_, the duration of the cathode pulse, t_on_, and the interval between pulses, t_off_; in reverse current, on the other hand, four parameters are required: cathode peak current density of the pulse, j_c_, the duration of the cathode pulse, t_c_, anode peak current density of the pulse, j_a_, and the duration of the cathode pulse, t_a_.

PC mainly used to influence the mechanisms of electrocrystallisation, which in turn control the mechanical and physical properties of the deposited metal^16,17^. Since the nucleation rate is proportional to the current density, the use of pulsed single or bipolar currents, which can reach densities significantly higher than their stationary counterparts, can produce deposits with reduced porosity and, a finer grain size^18,19^. During t_off_, when the current is interrupted, the ions of the active metal diffuse towards the electrode, where their concentration has decreased due to the deposition process. This process promotes the formation of new, smaller crystalline grains.

With PRC, during the anodic pulse, a partial redissolution of the deposited surface take place, which occurs mainly in the points with higher current density. Here there is greater deposition also in the cathodic phase, producing inhomogeneity in the thickness of the deposits. In this way the protrusions are smoothed with a consequent levelling effect of the metal surface^3,20^. In addition to this, throughout the electroplating process, a negatively charged state forms around the cathode. When DC is used, this electrodynamic diffusion layer is charged to a defined thickness, obstructing the flow of ions on the electrode surface^21,22^. In PC and PRC, this layer has the possibility of discharging (during t_off_ or t_a_), allowing ions to pass through the diffusion layer more easily and quickly.

PC deposition could reduce the need for additives up to 50–60%, while PRC offer the possibility to optimise of the stability and efficiency of the bath with negligible consumption of additives^23,24^. This could have positive effects in environmental terms, as it would reduce the polluting impact of galvanic wastes and the entire production process. The beneficial effects of non-stationary current are seen in the electrodeposition of both single metals and alloys, so that pulsed techniques are becoming increasingly popular in composite plating, amorphous alloys and anodizing^25^.

While there is a considerable amount of empirical information in the literature on how pulse plating variables affect the structure of the deposit, only a few systematic investigations have been carried out and the effects of current modulation on the microstructure of electrodeposited surfaces are still not well understood^26^. As a result of all these considerations, we decided to launch a preliminary study on everything related to the parameterisation of each deposition technique.

In this work we focussed on the acid copper bath because it is a ubiquitous solution in many industrial processes and has a quite simple composition. This type of bath has a stable and straightforward formulation that lends itself well to systematic analysis for new deposition techniques. In addition, industries use acid copper plating as an intermediate step in most electroplating processes^27,28^. Acid copper plating can improve subsequent electroplated layers adhesion and achieve a glossy finish^2^. Therefore, improvements in the copper deposition would have applications in many industrial processes. This study will be used as a first step for the application of pulsed current in the decorative field to more complex baths of precious metals and alloys. Once we chose the most promising parameters, samples were prepared and coated using both direct current and pulsed (PC) and reverse pulsed currents (PRC). The deposits obtained were characterised by estimating the metal surface’s deposition yield, homogeneity, hardness, and reflectivity.

## Materials and methods

We used a commercial acid copper electroplating bath (BLUCLAD 250 MUP) to replicate the industrial conditions as closely as possible. The solution consists in a full operative bath: 217.2 g/L CuSO_4_·5 H_2_O (0.87 M) as metal precursor, 66.1 g/L H_2_SO_4_ 98% (0.66 M) as electrolyte and all the necessary proprietary additives (brightening, levelling and complexing agent). This commercial acid copper plating bath has a working range between 3 and 5 A/dm^2^, we chose to use it within the upper limit of its characteristics to highlight any differences. Therefore, regardless of the type of current used, the average current density remains constant at 5 A/dm^2^, all tests were conducted without stirring.

The cell used, the renderings of which are shown in Supplementary Fig. S1, has dimensions 10 × 5 × 9 cm^3^ and was made of Moplen, an isotactic polypropylene thermoplastic material (PP-H), allowing us to drill a hole in one of its faces to create a housing for the cathode (cylinder of the chosen metal), so that the latter would face the surface to be coated inside the galvanic cell, but would still have one end outside that could be connected, via a clamp, to our electrical circuit. The anode was anchored to the face opposite the one presenting the hole for the cathode, so as to keep the distance between the two electrodes constant and reproducible throughout the experimental phases.

We used Pt electrode as a working electrode (diameter of 0.6 mm) when the electrochemical tests required an inert metal under the Cu deposit. Otherwise, we used Nickel electrodes with a diameter of 10 mm. Copper plate is always used as a counter electrode. A simple copper wire was chosen as a reference electrode because the solution used contains a very high and constant amount of copper ions. The value of this working electrode was compared with a commercial SCE electrode, obtaining a potential of 52 mV. Each test was carried out under controlled temperature conditions of 25 ± 1 °C.

XRF measurements were performed with a Bowman B Series XRF spectrometer (Schaumburg, IL, USA) using an acquisition time of 60 s, 50 kV tube voltage, 0.8 mA tube current, and a collimator of 0.6 mm in diameter. We used the spectra to obtain the thickness information with the FP method with one empirical point correction^29^.

Hardness was investigated through the Vickers Microhardness Meter (Microhardness Tester HX-1000) using a load of 10 g, the diamond tip was kept on the sample for a time of 15 s. The diagonals of the imprinted footprints give the hardness value in Vickers Pyramid Number **(**HV)^30^.

A DRS (Diffuse Reflectance Spectroscopy) spectrophotometer, Agilent Cary 300, was used to estimate the reflectance of each sample as a function of the wavelength of incident radiation. The high-resolution spectrophotometer used has an 8°/d geometry, with adaptive sample positioning and 7 mm diameter spots. Measurements were made between 380 and 780 nm with 5 nm steps.

Atomic force microscopy (AFM) measurements were performed in contact mode (0.5 V force set point) with a non-conductive Si_3_N_4_ triangular cantilever (Veeco, NP-S10, 0.12 N/m force constant) on a Molecular Imaging PicoSPM (10 μm × 10 μm, 512 px × 512 px, 1 lines/s speed) to evaluate the morphology and roughness of the samples.

Scanning electron microscopy (SEM–EDS) images were acquired using a Variable Pressure Hitachi SU3800 using an accelerating voltage of 5 kV.

## Results

### Pulsed current study

It is well known that good metal deposits are obtained in the frequency range from 10 to 500 Hz. It is also known that the range of valuable frequencies is limited by the capacitive effect of the double electric state at high frequencies and by mass transfer effects at low-frequency values, as shown in Supplementary Fig. S2. At high frequencies, the faradic current decreases approaching a state similar to that achieved in DC^31^. Similarly, we reach conditions close to DC at very low frequencies.

The quantitative criterion that mainly affects the best frequency range is the average potential, which increases both with the distortion of the faradic current wave at high frequencies and in the case of mass transfer limitations^32,33^. Hence the assumption that the minimum value of the average potential recorded indicates the frequency range for optimum deposition.

When working with pulsed current, we set the cathode current value, the cathode pulse time and the anode pulse duration, and the total electrodeposition period for each deposition. Equation 1 connects the three variables.

1 $$ J^{P} = J_{AV} \left( {P + 1} \right) $$

where J^P^ is the cathodic pulse current density; J_AV_ is the average current density; P is the pause/pulse ratio. We used the same average current density (5 A/dm^2^) for both pulsed and direct current deposition; for the value of P, we took measurements for t_off_/t_on_ ratios ranging from 1 to 5. The average current density value of the cathode pulse for each P are: P = 1, J^P^ = 10 (A/dm^2^); P = 2, J^P^ = 15 (A/dm^2^); P = 3, J^P^ = 20 (A/dm^2^); P = 4, J^P^ = 25 (A/dm^2^); P = 5, J^P^ = 30 (A/dm^2^).

**Figure 1:**
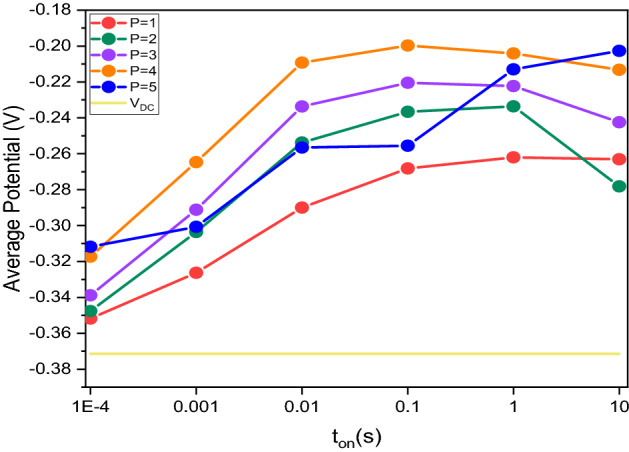
Average potential versus pulse time (t_on_), measured for each value of P at different pulse times (t_on_ = 10^−4^; 10^−3^; 10^−2^; 10^−1^; 1; 10 s) and for DC deposition.

For each value of P, we repeat the measurement with the following t_on_ times: 10^−4^; 10^−3^; 10^−2^; 10^−1^; 1; 10 s and the average potential value is recorded as reported in Fig. 1.

Since the literature defines, in some cases, ${\text{J}}_{{{\text{AV}}}} < {\text{J}}_{{\text{L}}}^{{{\text{DC}}}}$ and ${\text{J}}^{{\text{P}}} < {\text{J}}_{{\text{L}}}^{{\text{P}}}$ as the necessary operational limit, in others ${\text{ J}}^{{\text{P}}} < {\text{J}}_{{\text{L}}}^{{{\text{DC}}}}$ and ${\text{J}}^{{\text{P}}} < {\text{J}}_{{\text{L}}}^{{\text{P}}}$ (where ${\text{J}}_{{\text{L}}}^{{{\text{DC}}}}$ is the limiting current density under steady-state conditions and ${\text{J}}_{{\text{L}}}^{{\text{P}}}$ is pulse limit current density)^23,34^ we decide to calculate the values of ${\text{J}}_{{\text{L}}}^{{{\text{DC}}}}$ and ${\text{J}}_{{\text{L}}}^{{\text{P}}}$. The limiting current density value, ${\text{J}}_{{\text{L}}}^{{{\text{DC}}}}$, was obtained experimentally through potentiostatic measurements by performing a procedure of deposition and stripping in sequence, and the experimentally derived value is ${\text{J}}_{{\text{L}}}^{{{\text{DC}}}} = 17.5\;\frac{{\text{A}}}{{{\text{dm}}^{2} }}$.

Pulse limit current was derived through the Eq. (2):

2 $$ J_{L}^{P} = J_{L} \left( {\frac{{\delta^{P} }}{\delta }\left( {1 - \theta } \right) + \theta } \right)^{ - 1} $$

where δ^P^ is the pulsing diffusion layer, δ is Nernst diffusion layer and θ is the duty cycle (θ = 1/(P + 1)). All parameters respect the condition ${\text{J}}^{{\text{P}}} < {\text{J}}_{{\text{L}}}^{{\text{P}}}$, but only samples with a value of P = 1; 2 satisfy the operating limit ${\text{J}}^{{\text{P}}} < {\text{J}}_{{\text{L}}}^{{{\text{DC}}}}$. Based on these considerations, we decide to carry out two samples for each operational limit, for a total of four samples, whose parameters (pause/pulse ratio, cathode pulse, pause and cathode current value) are given below:

- P = 1; t_on_ = 0.01 s; t_off_ = 0.01 s; i^P^ =  − 0.0785
- P = 1; t_on_ = 0.1 s; t_off_ = 0.1 s; i^P^ =  − 0.0785
- P = 4; t_on_ = 0.01 s; t_off_ = 0.04 s; i^P^ =  − 0.1963
- P = 4; t_on_ = 0.1 s; t_off_ = 0.4 s; i^P^ =  − 0.1963.

The first two are more similar to those used in the literature and meet the conditions of both articles on limiting currents; the second two, on the other hand, are the best candidates according to Popov’s treatment^35–38^ and only meet the conditions of the first article on current limits.

### Reverse pulsed current

As far as reverse pulsed current is concerned, we proceed along two distinct paths: relying on the literature and on what are considered to be the optimal parameters for deposits with the characteristics we seek, but also starting from the criteria considered to be fundamental for reverse pulsed current electrodepositions and refining the setting based on the preliminary results we have obtained.

For the electrodeposition of copper, by sulphate and sulphuric acid solutions, without stirring, at room temperature, Vene and Nikolayeva^39^ claim that, irrespective of the overall current density and the period of the PRC wave, the best results were obtained with:

- $${\text{t}}_{{\text{a}}} /{\text{t}}_{{\text{c}}}$$ = 1/7; 3 $${\text{ t}}_{0}$$ < T < 16 $${\text{ t}}_{0}$$; $${\text{J}}_{{\text{a}}} /{\text{J}}_{{\text{c}}}$$ = 1

In solutions containing additives^1^, the optimum criteria for high penetration power are as follows:

- ${\text{t}}_{{\text{c}}} /{\text{t}}_{{\text{a}}}$ = 20; ${ }1 \le {\text{J}}_{{\text{a}}} /{\text{J}}_{{\text{c}}} \le 3.5$ (with the best value ${\text{J}}_{{\text{a}}} /{\text{J}}_{{\text{c}}}$ = 2); ${\text{q}}_{{\text{a}}} /{\text{q}}_{{\text{c}}} < 0.2$

Using the data considered ideal in the literature, we derive the parameters through theoretical considerations and practical results. Knowing the values of δ and D, we estimate t_0_ through the Eq. (3):

3 $$ t_{0} = \frac{{\delta^{2} }}{{\pi^{2} D}} = \frac{{\left( {5.8 \times 10^{ - 3} \;{\text{cm}}} \right)^{2} }}{{\pi^{2} \times 6.17 \times 10^{ - 6} \;\frac{{{\text{cm}}^{2} }}{{\text{s}}}}} = 0.55\;{\text{s}}{.} $$

The best depositions should occur for $$3 t_{0} \le T \le 16 t_{0}$$. Furthermore the Eq. (4) correlate the $$\frac{{t_{a} }}{{t_{c} }}$$ ratio with $$ t_{0}$$ and *T*.

4 $$ \frac{{t_{a} }}{{t_{c} }} = \left( {\frac{{4t_{0} }}{T}\ln \left( {\frac{2}{{1 + \exp \left( { - \frac{T}{{4t_{0} }}} \right)}}} \right)} \right)\left( {1 - \frac{{4t_{0} }}{T}\ln \left( {\frac{2}{{1 + \exp \left( { - \frac{T}{{4t_{0} }}} \right)}}} \right)} \right)^{ - 1} . $$

Combining the two expressions we obtain that $1.4 \le \frac{{t_{c} }}{{t_{a} }} \le 4.9$

We set an intermediate value to this range as a parameter for our electrochemical tests, namely:

- $${\text{t}}_{{\text{c}}} /{\text{t}}_{{\text{a}}}$$ = 3; $${\text{J}}_{{\text{a}}} /{\text{J}}_{{\text{c}}}$$ = 1

The anodic and cathodic current values are calculated using the same average current density as for the DC and PC tests, i.e. ${\text{J}}_{{\text{AV }}}$ = 5 A/dm^2^.

Knowing the ratios between t_c_/t_a_ and J_a_/J_c_, the individual currents can be calculated using the Eq. (5):

5 $$ J_{AV} = \frac{{q_{c} - q_{a} }}{{t_{c} + t_{a} }} = \frac{{J_{c} t_{c} - J_{a} t_{a} }}{{t_{c} + t_{a} }}. $$

We therefore have three different sets of variables concerning reverse pulsed current electrodeposition, which are reported below:

- $${\text{t}}_{{\text{a}}} /{\text{t}}_{{\text{c}}}$$ = 3; $${\text{J}}_{{\text{a}}} /{\text{J}}_{{\text{c}}}$$ = 1; i_c_ =  − 0.0771 A; i_a_ = 0.0771 A; q_a_/q_c_ = 0.33
- $${\text{t}}_{{\text{a}}} /{\text{t}}_{{\text{c}}}$$ = 7; $${\text{J}}_{{\text{a}}} /{\text{J}}_{{\text{c}}}$$ = 1; i_c_ =  − 0.0524A; i_a_ = 0.0524 A; q_a_/q_c_ = 0.14
- $${\text{t}}_{{\text{a}}} /{\text{t}}_{{\text{c}}}$$ = 20; $${\text{J}}_{{\text{a}}} /{\text{J}}_{{\text{c}}}$$ = 2; i_c_ =  − 0.0458 A; i_a_ = 0.0916 A; q_a_/q_c_ = 0.10

Based on the previously established deposition parameters, we evaluate the best cathodic and anodic pulse time to obtain homogeneous and brilliant deposits.

Even if the working conditions are different, we decide to proceed as we did for the pulse current. The potentiometric operating sequence is the same as that used for the pulsed current tests, with the only difference that for these samples, for each t_c_/t_a_ ratio of we have worked in sequence over the whole range of pulses, shown in Supplementary Table S1.

**Figure 2:**
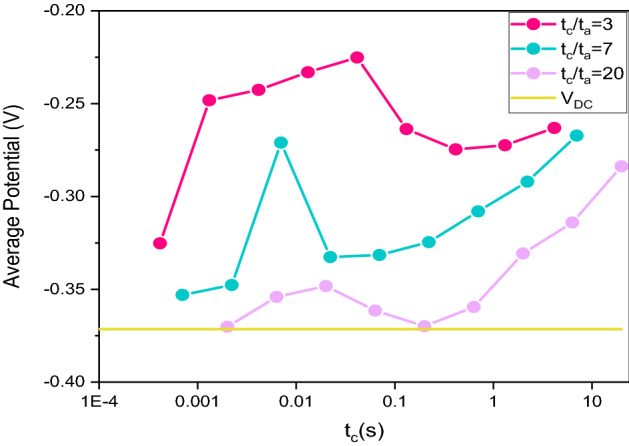
Average potential versus pulse time (t_on_), estimated for each t_c_/t_a_ ratio (t_c_/t_a_ = 3; 7; 20) at different cathode pulse times.

For each deposition, we extrapolated the average potential value, as reported in Fig. 2.

Based on the experimental values, we declare the following variables to be the best:

- $${\text{t}}_{{\text{c}}} /{\text{t}}_{{\text{a}}}$$ = 3; $${\text{J}}_{{\text{a}}} /{\text{J}}_{{\text{c}}}$$ = 1; $${\text{t}}_{{\text{c}}}$$ = 0.013123 s; $${\text{t}}_{{\text{a}}}$$ = 0.004269 s
- $${\text{t}}_{{\text{c}}} /{\text{t}}_{{\text{a}}}$$ = 3; $${\text{J}}_{{\text{a}}} /{\text{J}}_{{\text{c}}}$$ = 1; $${\text{t}}_{{\text{c}}}$$ = 4.15 s; $${\text{t}}_{{\text{a}}}$$ = 1.35 s
- $${\text{t}}_{{\text{c}}} /{\text{t}}_{{\text{a}}}$$ = 7; $${\text{J}}_{{\text{a}}} /{\text{J}}_{{\text{c}}}$$ = 1; $${\text{t}}_{{\text{c}}}$$ = 0.007 s; $${\text{t}}_{{\text{a}}}$$ = 0.001 s.

All prepared samples and their operating parameters (tipe of deposition current, cathode pulse duration, anode pulse duration, cathode and anode current values and frequency) are shown in Supplementary Table S2.

### Hardness

**Figure 3:**
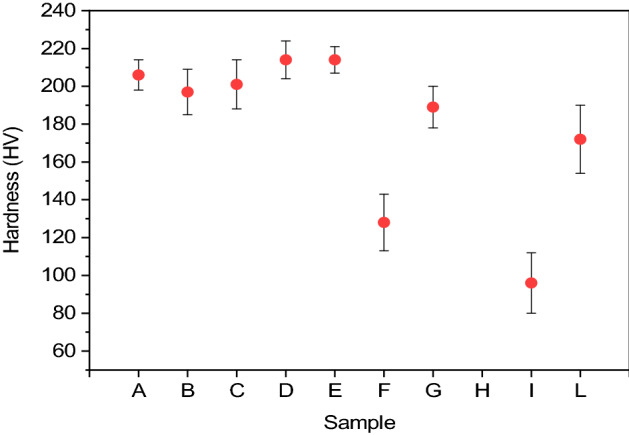
Hardness graph for each sample with relative standard deviations (excluding sample h for which measurement was not possible).

Hardness was investigated through the Vickers Microhardness Meter using a load of 10 g, the diamond tip was kept on the sample for a time of 15 s. The measurement was repeated on ten different random spots for each sample. For sample H, it was not possible to use the above parameters because the tip made no impression on the surface of the sample. Possible explanation derives from the surface morphology of the deposit, which is highly irregular, with clusters of deposition cores extending at least a few µm in height, hindering the penetration of the pyramidal diamond placed on the tip of the microdurometer. We report the average hardness and its standard deviation in Fig. 3.

The variation in the hardness of the film may be justified considering that modulated currents can change the compactness of the metal during electrodeposition processes; a further explanation may be that pulsed currents are able to influence the crystal structure of Cu in the nucleation and lattice growth phases^40–42^.

### Brightness

A colour characterisation is carried out for each sample whose images are shown in Supplementary Fig. S3. For each sample was estimated the reflectance value using a UV–Vis spectrophotometer. We decide to evaluate as a parameter the relative reflectance of each sample, compared to sample A obtained in DC, since the latter is the technique considered as the state of the art in electroplating. The reflectance values obtained using the UV–Vis spectrophotometer are first normalised against the reflectance of black and white using the Eq. (6):

6 $$ R_{C}^{N} = \frac{{R_{C} - R_{N} }}{{R_{B} - R_{N} }}. $$

And then compared to sample A with the Eq. (7):

7 $$ R\% = \frac{{R_{C}^{N} - R_{A}^{N} }}{{R_{A}^{N} }} \times 100. $$

**Figure 4:**
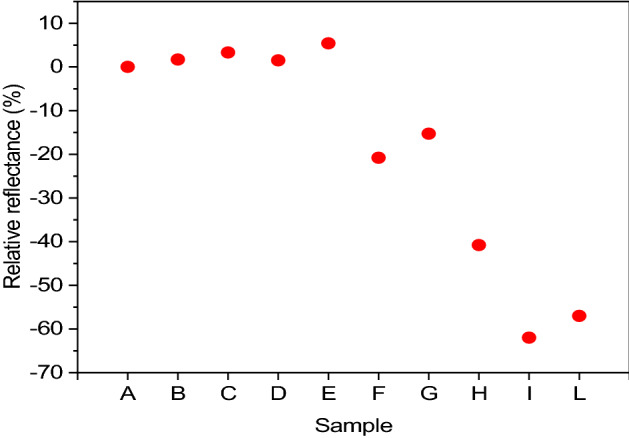
Relative reflectance of each sample to A estimated by UV–Vis spectrophotometry.

The results obtained are reported in Fig. 4.

Since reflectance values are closely related to the surface morphology of the samples, we performed SEM measurements to observe the metal films at microscopic scale, as shown in Supplementary Fig. S4. From the results it is evident that samples from A to E (DC and pulse current samples) present very small crystals, leading to an extremely smooth and compact surface. To emphasise the morphology of these samples the contrast of the images was maximised, inevitably losing quality.

In order to appreciate the surface of the samples in even greater detail, AFM analysis was performed on the sample that showed a higher reflectance value (sample E) compared to the sample produced using direct current (sample A). As shown in the 3D images in Supplementary Fig. S5, the sample produced using pulsed current (E) showed lower roughness value (Sq = 7.41 nm) than the reference sample A (Sq = 13.71 nm).

### Bath efficiency

The efficiency of the electroplating bath is estimated by gravimetric analysis and the actual thickness of the metal film was obtained from the empirical mass; the results obtained are reported in Table 1.

**Table 1:** Percentage efficiency of each electroplating bath estimated by gravimetric analysis.

Sample	Bath efficiency (%)	Sample	Bath efficiency (%)
A	95.4 ± 0.3	F	95.3 ± 0.3
B	97.9 ± 0.3	G	98.5 ± 0.3
C	99.0 ± 0.3	H	92.3 ± 0.3
D	99.3 ± 0.3	I	98.9 ± 0.3
E	98.2 ± 0.3	L	86.8 ± 0.3

### Inhomogeneity

**Figure 5:**
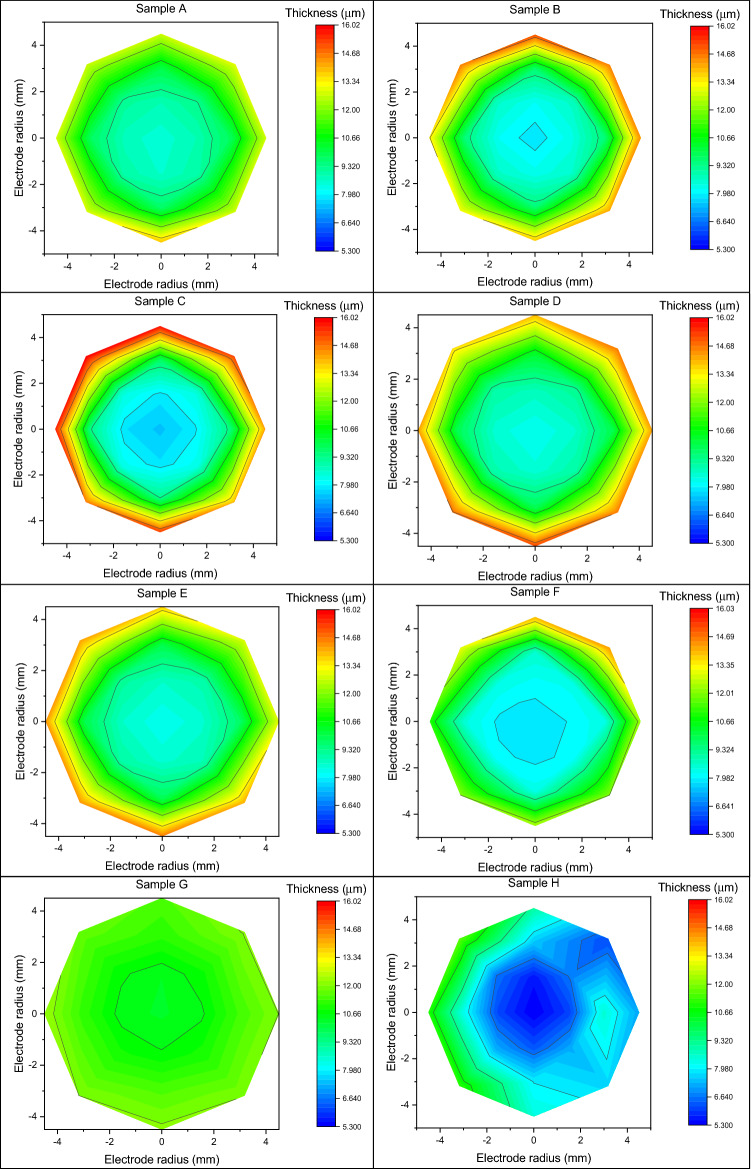

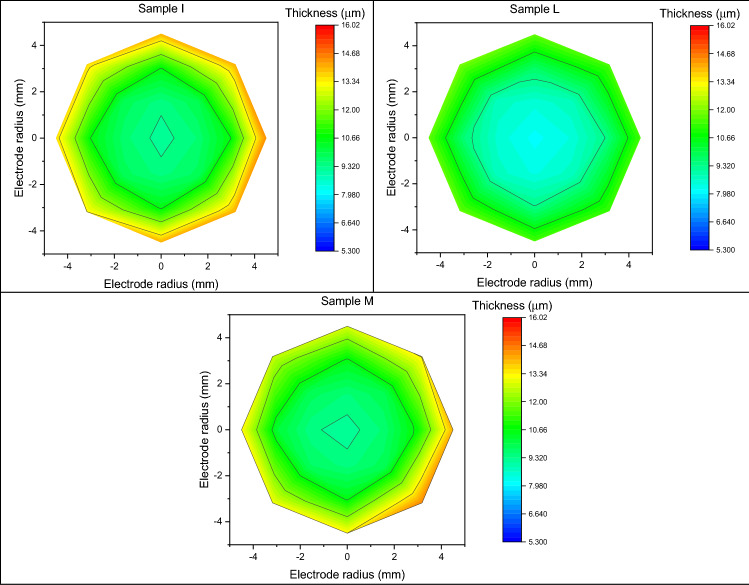
Graphic representation of the thicknesses by colour scale of each sample.

Through X-ray fluorescence spectroscopy, we probe the thickness of the sample. We created a mapping grid to estimate the thickness of the deposit by measuring 21 points arranged in a radial geometry with respect to the centre of the sample, of which a graphic representation of the thicknesses by colour scale is shown in Fig. 5.

The average thickness of the Cu deposit, measured at the centre of the samples, is 8.11 ± 0.41 µm; the sample that showing the lowest thickness is H with a deposit height of 5.30 ± 0.27 µm, while G is the sample with the highest thickness (10.40 ± 0.52 µm).

The measured metal film heights for each sample are fitted using an exponential function having the Eq. (8):

8 $$ y = a + b \left( {e^{x/c} + e^{ - x/c} } \right). $$

In this case, we do not consider the standard deviation (reported in Eq. (9)) as the statistical error on thickness but as an estimator of deposition uniformity. A higher value of standard deviation corresponds to a less homogeneous deposit; the results are reported in Table 2.

9 $$ \sigma = \sqrt {\frac{{\mathop \smallint \nolimits_{0}^{r} 2\pi r\left( {f\left( r \right) - \overline{h}} \right)^{2} dr}}{A}} . $$

**Table 2:** Standard deviation, considered as an estimator of deposit uniformity, relative to each sample.

Sample	Inhomogeneity (µm)	Sample	Inhomogeneity (µm)
A	1.84	F	2.58
B	2.69	G	0.53
C	3.30	H	1.14
D	2.59	I	1.31
E	2.37	L	1.71

## Discussion

We can summarise the results obtained, through the various characterisations, concerning the quality and performance parameters investigated, for each sample, in Table 3.

**Table 3:**
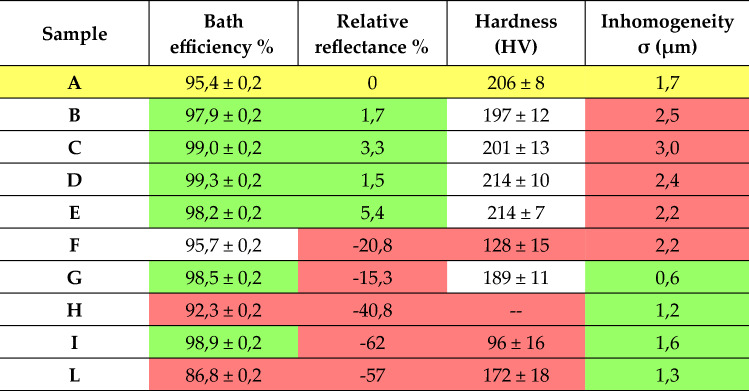
Summary of characterisations and results obtained.

In yellow the reference values obtained for the DC sample; in red the results showing a worsening compared to those obtained in DC and in green the improved ones.

For all the results obtained, relative to each parameter investigated, the reference value is that estimated for sample A, the result of a direct current electrochemical process, which is, to date, the most widely developed and used technique in the industrial field for the most varied electroplating processes. Therefore, the parameters to be equalled and, if necessary, exceeded in performance are highlighted in yellow. The better values compared to those achieved with the DC sample are highlighted in green; in red are those for which there is a worsening; in white are the parameters that can be considered comparable (considering the error) to those of A, for which there is no significant difference in quality compared to DC.

It is evident that all the samples produced using pulsed current show comparable characteristics, if not superior, to those of the A deposit, in almost all the characterisations carried out, except for homogeneity, which appears to have deteriorated. The parameterisation that proves to be most effective in producing a fine-grained, homogeneous and compact deposit using pulsed current is the one for which the ratio between the pause time and the pulse time is equal to 1, with pulse duration ranging from 0.01 (sample D) to 0.1 s (sample E); under these conditions, the efficiency of the bath also proves to be optimal. Both samples with P = 1 were also those that met the most stringent conditions regarding the pulse current density, i.e. ${\text{J}}^{{\text{P}}} < {\text{J}}_{{\text{L}}}^{{{\text{DC}}}}$ and ${\text{J}}^{{\text{P}}} < {\text{J}}_{{\text{L}}}^{{\text{P}}}$.

The only negative deviation, although not high, is with the average thickness, which is not uniformly distributed but affected by the edge effect; for sample E, however, this aspect is minimal compared to the others.

Concerning the deposits obtained in reverse pulsed current, no particular improvement in the quality of the metal film obtained can be seen, other than that of a highly homogeneous surface, thanks to the intrinsic characteristics of the deposit in bipolar current, which has a levelling effect thanks to the anodic pulses. This is true for all the samples, except for one, sample F, which has ${\text{t}}_{{\text{c}}} /{\text{t}}_{{\text{a}}}$ = 7 and ${\text{t}}_{{\text{c}}}$ = 7 s; probably the pulse times (especially the cathodic one) are too long, so much so that they tend to a stationary condition as if at an electrochemical level two direct currents were alternately used and no longer a reverse pulsed current. We can make a similar consideration for the other sample (H) obtained with pulses in the second's range, i.e. ${\text{t}}_{{\text{c}}} /{\text{t}}_{{\text{a}}}$ = 3 and ${\text{t}}_{{\text{c}}}$ = 4.15 s, which is worse than A in all characterisations except for the homogeneity of the deposit. The other sample (L) with a ratio of cathodic to anodic pulse times equal to 7 also shows unsatisfactory results, leading us to believe that precisely the parameterisation of the reverse current is not optimal, at least for this acid coppering bath. The best setting, among the various parameterisations of the bipolar pulses, appears to be the one used for sample G, i.e. ${\text{t}}_{{\text{c}}} /{\text{t}}_{{\text{a}}}$ = 20 and ${\text{t}}_{{\text{c}}}$ = 0.02 s. Although the results are not comparable to those obtained with direct current or even pulsed current, it may be preferred in technical applications for which the homogeneity of the deposit is more important than its aesthetic appearance.

## Conclusions

In this preliminary study on modulated current electrodeposition, we did not succeed in obtaining a copper deposit that presents improvements on all the qualities investigated.

Samples produced with pulsed current show improved characteristics compared to those obtained with direct current. This is especially true for the finishing of the piece, as the deposits are more compact and finer-grained. In almost all samples the metal films are shinier and more mirror-like.

The samples obtained using pulsed reverse currents, on the other hand, show a particular uniformity of deposit. Although the finish of the metal films is not brilliant, the thickness of the deposits is extremely homogeneous over the entire surface.

In conclusion, the optimal parameters were identified to obtain a compromise between the specific characteristics according to those desired for the industrial processing that the plated object will undergo. Based on the results obtained, pulsed currents will be preferred in electroplating applications where an optimum finish of the plated item is required. The process can be applied in fashion, furniture and all those sectors involving multilayer depositions, where the acid copper coating is used to obtain a good levelling, smooth and shiny surface. Reverse pulsed currents are recommended for applications where aesthetics is negligible but is required a good homogeneity of metal thickness. Thanks to the negative current transient, the deposit undergoes a process of self-levelling caused by the preferential redissolution of the metal film in the parts with higher surface energy; this feature could be useful in all those electroplating contexts where printed circuit boards are produced.

## Supplementary Information

Supplementary Information.

## Data Availability

The generated and analysed data during the current study is supplied in this manuscript and supplementary material. To access the data, all can contact Massimo Innocenti (minnocenti@unifi.it).
