# Supplementary material for: A systematic study of pulse and pulse reverse plating on acid copper bath for decorative and functional applications
Source: Sci Rep. 2022 Oct 28;12:18175. doi: 10.1038/s41598-022-22650-x (PMC9616928; doi:10.1038/s41598-022-22650-x)
Supplement: Supplementary file 1 — Supplementary Information. [file 41598_2022_22650_MOESM1_ESM.docx]

**SUPPLEMENTARY INFORMATION**

A systematic study of pulse and pulse reverse plating on acid copper bath for decorative and functional applications

Elena Mariani ^1^, Walter Giurlani ^1, 2,^ *, Marco Bonechi ^1,2^, Vincenzo Dell’Aquila ^3^ and Massimo Innocenti ^1, 2, 4, 5,^ *

^1^ Dipartimento di Chimica, Università degli Studi di Firenze, via della Lastruccia 3, 50019 Sesto Fiorentino, Italy; elena.mariani@unifi.it (E.M.)

^2^ INSTM, Consorzio Interuniversitario Nazionale per la Scienza e Tecnologia dei Materiali, via G. Giusti 9, 50121 Firenze, Italy

^3^ Eco-Tech Finish s.r.l., Z.I. San Zeno, Strada C 27, 52100 Arezzo, Italy

^4^ CNR-ICCOM, Istituto di Chimica dei Composti OrganoMetallici, via Madonna del Piano 10, 50019 Sesto

Fiorentino (FI), Italy

^6^ CSGI, Center for Colloid and Surface Science, via della Lastruccia 3, 50019 Sesto Fiorentino, Italy

* Correspondence: walter.giurlani@unifi.it (W.G.); [m.innocenti@unifi.it](mailto:m.innocenti@unifi.it) (M.I.)

| $\mathbf{t}_{\mathbf{c}}\mathbf{/}\mathbf{t}_{\mathbf{a}}$ **= 3** | | $\mathbf{t}_{\mathbf{c}}\mathbf{/}\mathbf{t}_{\mathbf{a}}$**= 7** | | $\mathbf{t}_{\mathbf{c}}\mathbf{/}\mathbf{t}_{\mathbf{a}}$**= 20** | |
| --- | --- | --- | --- | --- | --- |
| $\mathbf{t}_{\mathbf{c}}$ **(s)** | $\mathbf{t}_{\mathbf{a}}$ **(s)** | $\mathbf{t}_{\mathbf{c}}$ **(s)** | $\mathbf{t}_{\mathbf{a}}$ **(s)** | $\mathbf{t}_{\mathbf{c}}$ **(s)** | $\mathbf{t}_{\mathbf{a}}$ **(s)** |
| 0,000415 | 0,000135 | 0,000700 | 0,000100 | 0,002000 | 0,000100 |
| 0,001312 | 0,000427 | 0,002214 | 0,000316 | 0,006325 | 0,000316 |
| 0,004150 | 0,001350 | 0,007000 | 0,001000 | 0,020000 | 0,001000 |
| 0,013123 | 0,004269 | 0,022136 | 0,003162 | 0,063246 | 0,003162 |
| 0,041500 | 0,013500 | 0,070000 | 0,010000 | 0,200000 | 0,010000 |
| 0,131235 | 0,042691 | 0,221359 | 0,031623 | 0,632456 | 0,031622 |
| 0,415000 | 0,135000 | 0,700000 | 0,100000 | 2,000000 | 0,100000 |
| 1,312345 | 0,426907 | 2,213590 | 0,316228 | 6,324555 | 0,316228 |
| 4,150000 | 1,350000 | 7 | 1 | 20 | 1 |

**Supplementary Table S1.** Cathode pulse duration (t_c_) and anode pulse duration (t_a_) for each of the three parameter sets (t_c_/t_a_ = 3; 7; 20)

| **Name** | **Current** | $\mathbf{t}_{\mathbf{c}}$ **(s)** | $\mathbf{t}_{\mathbf{a}}$ **(s)** | $\mathbf{i}_{\mathbf{c}}$ **(A)** | $\mathbf{i}_{\mathbf{a}}$ **(A)** | **F (Hz)** |
| --- | --- | --- | --- | --- | --- | --- |
| **A** | **DC** | 600 | - | -0,0392 | - | - |
| **B** | **PC** | 0,1 | 0,4 | -0,1963 | 0 | 2 |
| **C** | **PC** | 0,01 | 0,04 | -0,1963 | 0 | 20 |
| **D** | **PC** | 0,01 | 0,01 | -0,0785 | 0 | 50 |
| **E** | **PC** | 0,1 | 0,1 | -0,0785 | 0 | 5 |
| **F** | **PRC** | 7 | 1 | -0,0524 | 0,0524 | 0,125 |
| **G** | **PRC** | 0,02 | 0,001 | -0,0458 | 0,0916 | 48 |
| **H** | **PRC** | 4,15 | 1,35 | -0,0771 | 0,0771 | 0,182 |
| **I** | **PRC** | 0,013123 | 0,004269 | -0,0771 | 0,0771 | 57,498 |
| **L** | **PRC** | 0,007 | 0,001 | -0,0524 | 0,0524 | 125 |

**Supplementary Table S2.** Totality of samples prepared and their operating parameters (type of deposition current, cathode pulse duration, anode pulse duration, cathode and anode current values)

| 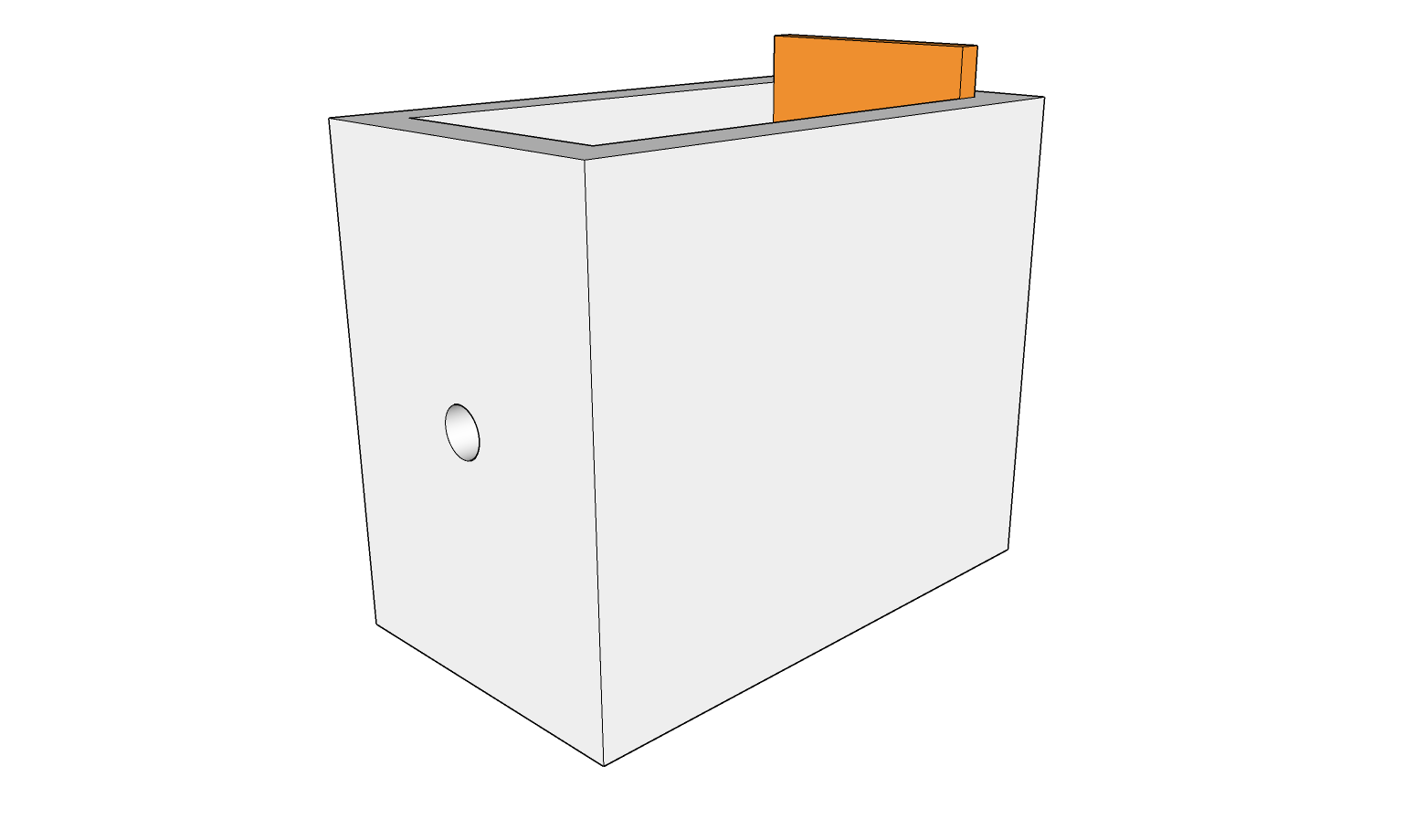 | 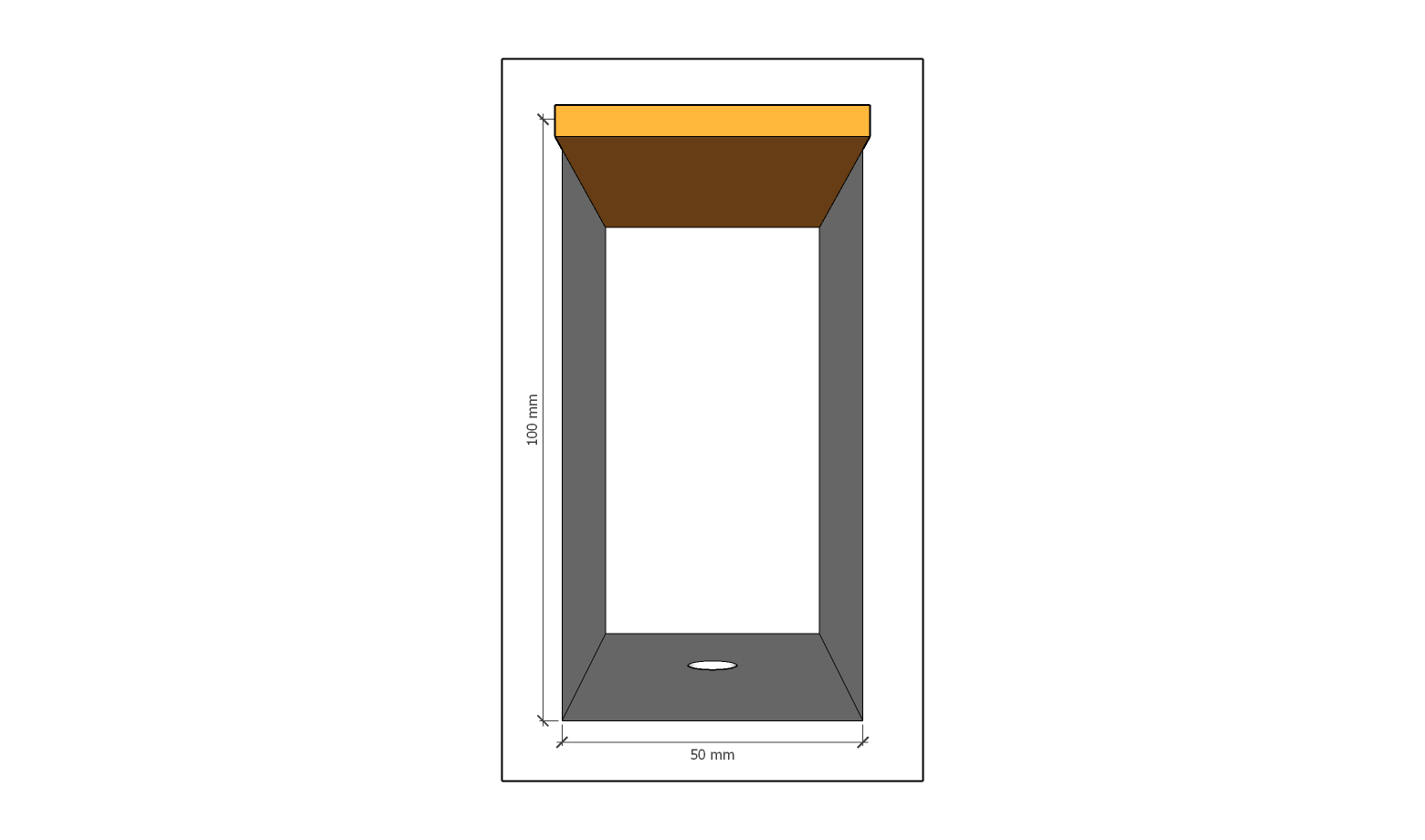 |
| --- | --- |

**Supplementary Figure S1:** Rendering of the galvanic cell used for the experiments

| **** | **** | **** |
| --- | --- | --- |

**Supplementary Figure S2**: Examples of potential-time curves for P=2 in which the potential is influenced by the pulse time. **a**) t_on_ = 10^-4^ s: for pulsed that are too short, the potential suffers from the capacitive effect, never being able to reach steady state. **b**) t_on_ = 0,01 s: the deposition process is only controlled by activation kinetics. **c**) t_on_ = 10 s: for pulses that are too long the potential suffers from the depletion by ions in the solution adjacent to the electrode, entering in a diffusive regime

| **Sample A**  **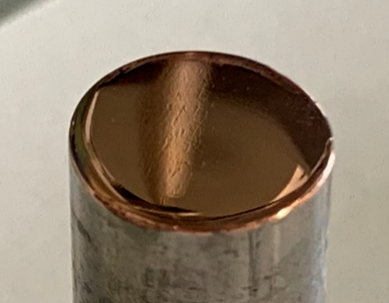** | **Sample B**  **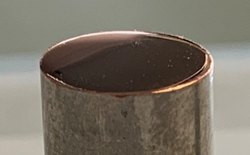** | **Sample C**  **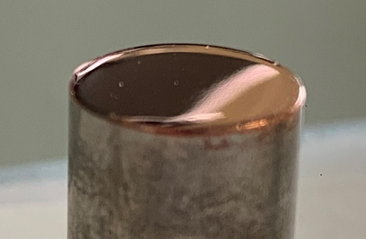** | **Sample D**  **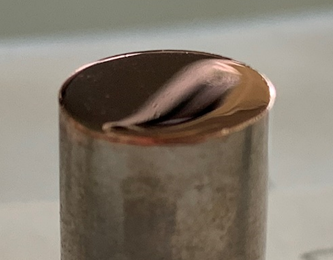** |
| --- | --- | --- | --- |
| **Sample E**  **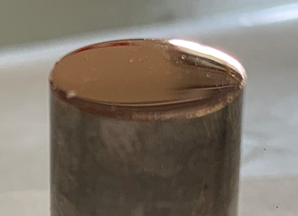** | **Sample F**  **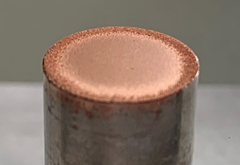** | **Sample G**  **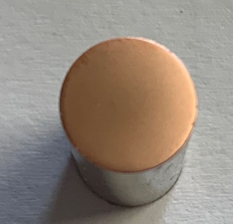** | **Sample H**  **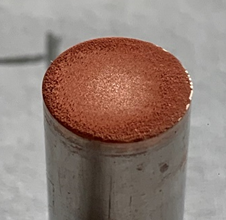** |
| **Sample I**  **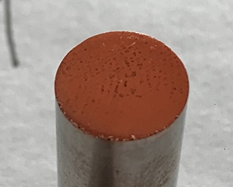** | | **Sample L**  **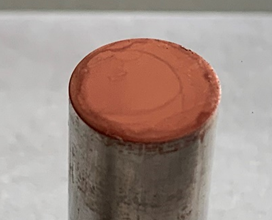** | |

**Supplementary Figure S3.** Samples at the end of each electrodeposition process

| **Sample A**  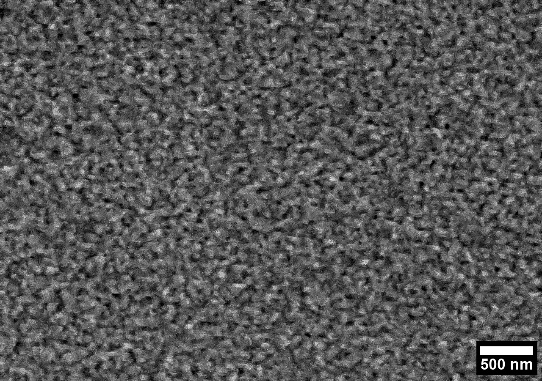 | **Sample B**  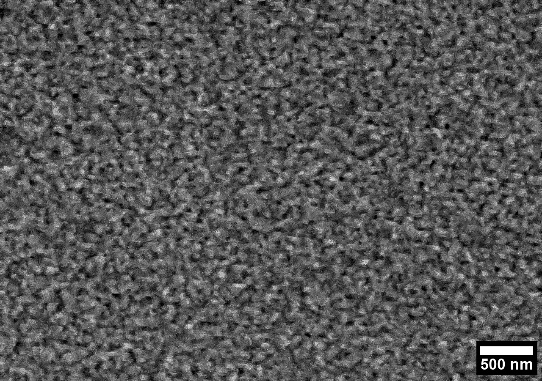 | **Sample C**  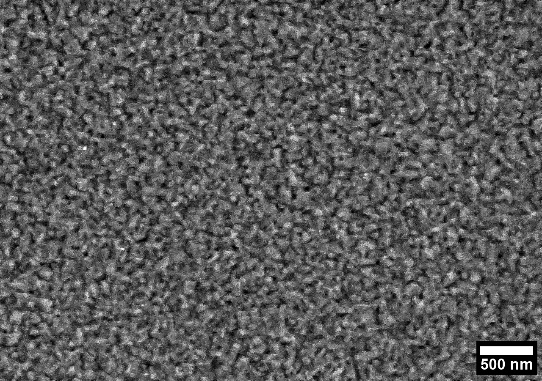 |
| --- | --- | --- |
| **Sample D**  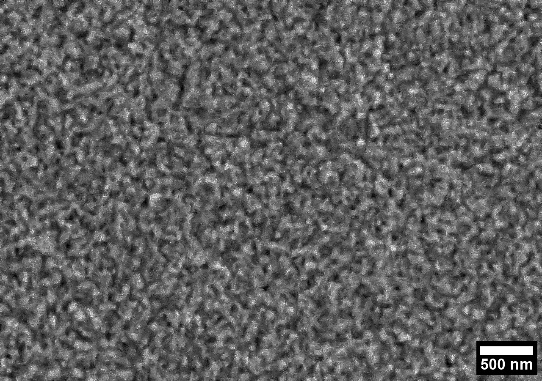 | **Sample E**  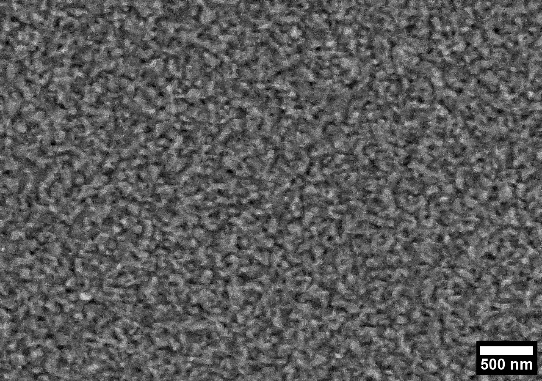 | **Sample F**  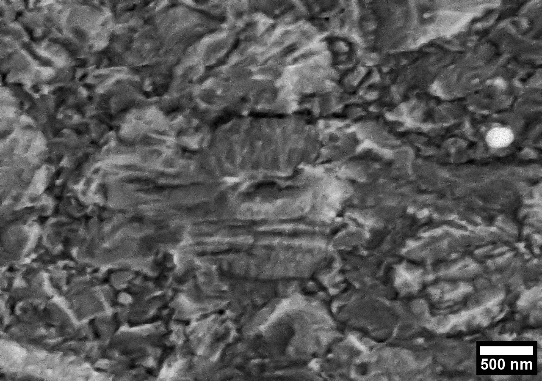 |
| **Sample G**  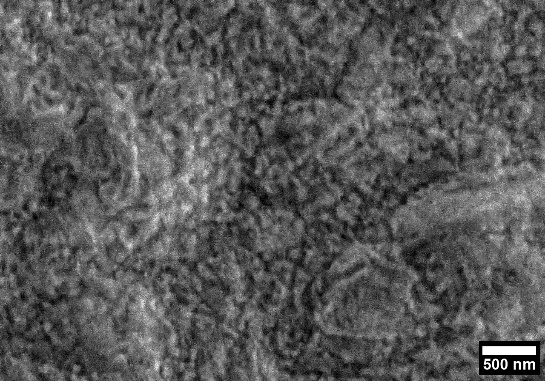 | **Sample H**  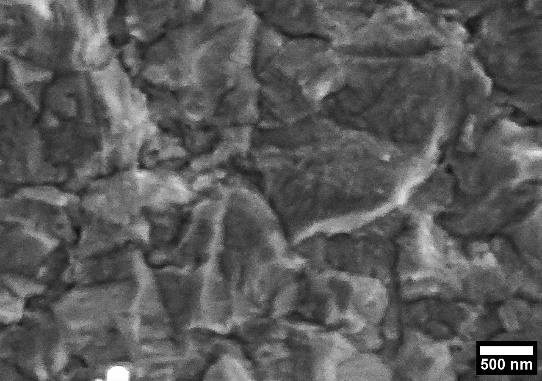 | **Sample I**  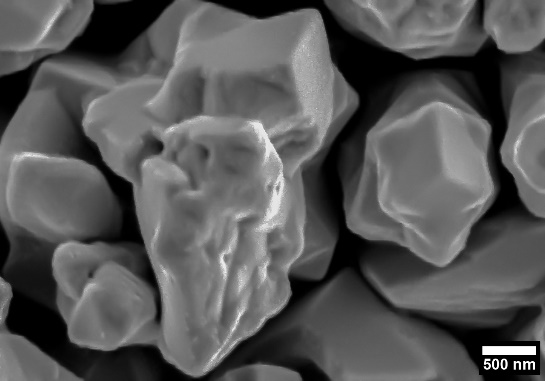 |
| **Sample L**  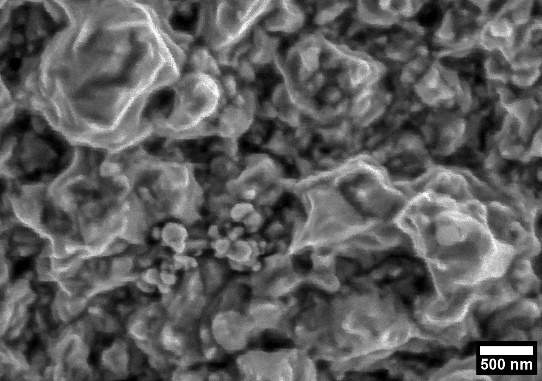 | | |

**Supplementary Figure S4.** SEM images of the electrodeposited Cu surface of each sample

| **Sample A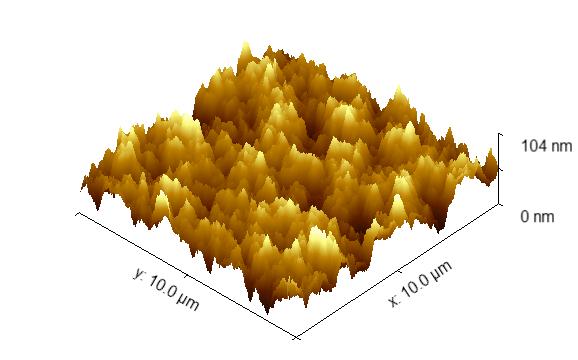** | **Sample E**  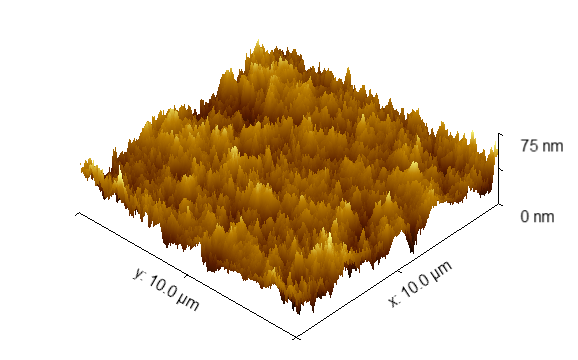 |
| --- | --- |

**Supplementary Figure S5.** Comparison between surfaces AFM images of sample A (DC) and sample E (PC)
